# New York State Veterinary Medical Society

**Published:** 1892-12

**Authors:** N. P. Hinckley

**Affiliations:** Secretary, Buffalo, N. Y.


### EDITORIAL.

### NEW YORK STATE VETERINARY MEDICAL SOCIETY.

The New York State Veterinary Medical Society will hold its third annual meeting at the parlor of the Vanderbilt House, Syracuse, N. Y., on Thursday, January 12th, 1893.

The following subjects will be reported for discussion: "Influenza and its complications;" "Antiseptics, their uses and mode of application;" "Tuberculosis, especially its generation."

Reference to the September Number of the JOURNAL will show how active and interesting the work of this Society has become, and it is imperative for the honor of the Empire State, as well as for the personal benefit of each veterinarian in it, that the ranks of the Society shall be filled by all reputable practitioners of the State, and that they shall attend the meetings, and place them among the best in the country.

Next year the United States Veterinary Medical Association will receive many eminent foreign veterinarians. The New York State Veterinary Medical Society must prepare to take its proper place in the representation of the profession in this country, and will probably individually desire to receive visitors at home.

No member should fail to attend the meeting on January 12th. All reputable veterinarians are cordially invited to attend and take part in the proceedings.

For detailed information, and blanks for application for membership, address

N. P. HINCKLEY, D.V.S., *Secretary*,
Buffalo, N. Y.

